# Paternal Relatedness Predicts the Strength of Social Bonds among Female Rhesus Macaques

**DOI:** 10.1371/journal.pone.0059789

**Published:** 2013-03-20

**Authors:** Oliver Schülke, Svenja Wenzel, Julia Ostner

**Affiliations:** Courant Research Centre Evolution of Social Behaviour, Georg August University Göttingen, Göttingen, Germany; Max Planck Institute for Evolutionary Anthropology, Germany, Germany

## Abstract

Forming strong, equitable, and enduring social bonds with a few individuals in a group carries adaptive benefits in terms of increased longevity, offspring survival and paternity success in birds and mammals, including humans. These recent insights generated a new interest in the factors creating variation in the strength of social relationships. Whether and how animals discriminate paternal kin from non-kin and bias their social behavior accordingly is being debated. This study explores the relative importance of dominance rank, age, maternal and paternal relatedness in shaping dyadic affiliative relationships in a group of 30 captive rhesus macaque females. The strength of social relationships, measured by the composite sociality index from observational data, was independently predicted in GLMMs by both maternal and paternal relatedness as well as rank similarity. In addition, social bonds between paternal half-sisters were stronger than between distantly related kin suggesting that females biased their affiliative effort towards paternal relatives. Despite identical relatedness coefficients bonds between mothers and their daughters were three times as strong as those between full sisters. Together these results add to the growing body of evidence for paternal kin biases in affiliative behavior and highlight that females prefer descendent over lateral kin.

## Introduction

Gregarious animals form social relationships with group members they repeatedly engage with if past interactions influence future ones [1]. Such relationships can be highly differentiated within a group, i.e. the majority of relationships are weak and each individual only forms a few very strong positive relationships characterized by frequent affiliation and frequent association in close spatial proximity [2], [3]. Evidence is mounting that such strong affiliative relationships or friendships [2] affect individual fitness in mammals [4], [5]. The strength and stability of social bonds but not their number predict female fertility, offspring survival [6], [7], [8], female longevity [9], [10], and male reproductive success [11]. These fitness benefits can be enormous, for example in female baboons (*Papio sp.*) where females with bonds of intermediate strength experience on average a 50% increase in reproductive life span compared to females with weak bonds [9]. In light of this strong selection pressure [12] it becomes increasingly important to further our understanding of the factors that determine and maintain the variation in the strength of social bonds. Here we present a study of female affiliative relationships in rhesus macaques (*Macaca mulatta*) considering the effects of dominance rank distance, age similarity, maternal, and paternal relatedness.

In species with linear dominance hierarchies rank distance structures social relationships and closely ranked individuals develop stronger bonds than more distantly ranked ones [13], [14], [15]. This relation may be a consequence of competition for high ranking partners leading to a concentration of affiliative behavior in rank neighbors when the number of partners or the time the dominants have available for affiliative exchanges is constrained [16] or a general preference for closely ranked individuals [13]. Alternatively, the social bond between individuals may in fact be the first step, with closely bonded partners supporting each other in agonistic conflicts and pulling each other towards similar dominance ranks as a result. This latter explanation has been put forward as the fundamental mechanism underlying matrilineal dominance hierarchies [17], [18] but has also been found in dispersing male macaques [11].

The effect of rank distance on the strength of social bonds has been shown to be independent of maternal relatedness among partners [13], [19] but the size of the rank distance effect may vary with the demography of the group [20] and type of kinship measure used [21]. Maternal relatedness generally has very strong effects on the strength of social bonds [19], [22], [23], [24], [25], [26], [27]. Bonds between mothers and their offspring that are maintained into adulthood [28] are the strongest bonds found in the animal kingdom [5], usually followed by those between maternal sisters. More distant maternal kin, such as aunts and nieces, may still form strong bonds, but cousins tend not to be more closely bonded than non-kin [21], [25], [29], [30].

The effect of paternal relatedness on social bonds is less well understood [31], partly owing to the difficulty of assessing paternal relatedness in multimale-groups with promiscuous mating systems. However, female rhesus macaques have been shown to form closer bonds with paternal sisters than non-kin [24], [25] and to aggressively target paternal sisters less frequently than unrelated females [32]. Female baboons (*Papio cynocephalus)* form slightly closer bonds with paternal sisters than with unrelated females [27], [33]. Yet chimpanzee males (*Pan troglodytes*, [34]), sun-tailed monkeys (*Cercopithecus solatus* [22]), and female capuchin monkeys (*Cebus capucinus* [20]) do not differentiate between paternal siblings and unrelated group members in the strength of relationships formed while results from juvenile mandrills (*Mandrillus sphinx*) are inconclusive [35].

In humans age similarity is a major predictor of friendship which is thought to result from shared interests promoting joint activities that strengthen ties among closely aged individuals. Among animals age difference is often used to investigate whether age similarity is used as a cue for paternal relatedness in species with high male reproductive skew and short tenure, i.e. where one or a few males sire all offspring over a short period of time with sires changing across time [19], [36]. Captive studies have produced mixed results but recent large-scale studies which controlled for age differences still detected paternal kin effects [24], [31], [35].

Here we aim at providing additional data to the understudied role of paternal kinship in shaping animal social structure. Following the most comprehensive treatments of the issue [24], [27], [34] we test the independent effects of age difference, rank difference as well as maternal and paternal relatedness on the strength of social bonds between adult female rhesus macaques. In addition we test whether the strength of social bonds differs between certain kin categories. We specifically predict that social bonds will be stronger between paternal half-sisters than between distantly related females. We also predict that if paternal relatedness is assessed by the macaques with less precision than maternal relatedness, social bonds between mothers and daughters will be stronger than those between full-sisters although total relatedness is 0.5 in both cases.

## Methods

The paper reports results from a strictly non-invasive and purely observational study on rhesus macaques. Data were collected from all females (21 adults and 9 juveniles, the latter were sexually mature but observed before their first parturition) from a breeding group of 66 rhesus macaques housed at the German Primate Center Göttingen, Germany. The macaques live in a large social group in a system of two large indoor (40sqm each) and one very large outdoor enclosure (approximately 280sqm). The monkeys have ad libitum access to water from several faucets distributed across the enclosure to avoid competition for access to water. Keepers provide changing devices for environmental enrichment and monkeys are fed three times a day (monkey chow in the morning, porridge from different grains with vitamins, minerals and milk products added around noon, and fresh fruit and vegetables, monkey chow and grains in the afternoon). Routines at the facility are in agreement with EU-Guideline 2010/63/EU for the protection of animals used for scientific research and the German Protection for Animals Act. The observational study was approved by the German Primate Center and did not interfere with the regular routines of husbandry. As such, no permits were required for the procedures used.

Behavioral observations were carried out using 15 min focal animal protocols [37] of continuously recorded agonistic (bite, slap, push-pull, lunge, chase, make-room, crouch, give ground, bare teeth) and affiliative interactions (mainly grooming and contact sitting) as well as approaches into and retreats from close proximity of 1m. Females were observed in a randomized predetermined sequence for a total of 3 hours 40 min each. Additional data on the outcome of decided agonistic conflicts were collected ad libitum by SW and a second observer.

A dominance hierarchy was constructed based on the outcome of 1098 dyadic decided agonistic conflicts (spontaneous submission or submission only as a response to aggression from the opponent) using the I&SI method as implemented in MatMan1.2© (Noldus, Wageningen, The Netherlands). The hierarchy was significantly linear when tested against 10,000 randomized matrices at h’ = 0.74 and p<0.0001. Eighteen percent of relationships were unknown, 4% two-way, 2% tied, and 2% inconsistent. The directional consistency index of winning was high at 0.96.

The strength of social bonds was assessed using the Composite Sociality Index CSI [27] and based on three specific affiliative behaviors, i.e. staying in close spatial proximity without explicit affiliative contact, friendly body contact without allo-grooming, and allo-grooming. Any two of the three components were highly correlated across all dyads in the group (average row-wise rho between 0.61 and 0.79 in row-wise matrix correlations with 10,000 permutations using Spearman correlations). To calculate the CSI we divided the rate of interaction for each specific behavior for each dyad by the average rate across all dyads, then added them up and divided by three for the three behaviors. The mean of the CSI across all dyads by definition is 1, consequently, values above 1 indicate above average bond strength, and values below 1 a weak bond [27].

Paternity of all females was known as only one of a total of four different mature males lived with the group at any given time and the founder females were coming from different sources. Maternal relatedness had been verified with genetic relatedness analyses in the Department of Genetics of the German Primate Center Göttingen (unpubl. data DPZ Göttingen). For the analyses relatedness was derived from complete pedigrees assuming relatedness between each parent and its direct offspring to be 0.5. The only kin relation on the paternal side was paternal half-sister-ship. Either fathers or offspring were moved to another enclosure before they could breed in the same group. If paternal sisters were also related maternally at r ≥0.0625 we excluded the dyad from all comparisons between kin categories. Kin relations on the maternal side were more diverse, including mother-daughter, half-sister, aunt-niece, cousin, grandmother-granddaughter, great aunt-great niece, as well as distant aunt-niece and cousin relations up to the 3rd degree (r = 0.00391). When deriving r-values for the different dyads care was taken as to whether the last common relation was a unilateral or a bilateral one, e.g. when two females’ mothers were full sisters the females were cousins with r = 0.125, but when the mothers were only half-sisters the cousins were related at r = 0.0625 only. In many dyads females were related several different ways, e.g. as aunt and niece but also as 2nd degree cousins and r-values for the different relations were added up to give the total relatedness between two females (here 0.125+0.015625 = 0.140625). Bonds between mother-daughter dyads and different types of sisters were all stronger (also p<0.05 in permutation tests described below) than between aunts and nieces (r = 0.125, because mother is only half-sister of aunt). Therefore, females were classified as “distant kin” when their combined maternal and paternal relatedness coefficient was ≤0.125.

To test whether CSI values were influenced by maternal and/or paternal relatedness, age difference or rank difference we built general linear mixed models [38] into which we included up to four predictor variables (absolute ordinal rank distance, absolute age difference, maternal coefficient of relatedness, and whether females were paternal sisters or not) as fixed effects and the two individuals involved as two categorical random effects. The models were fitted in R [39] using the function lmer of the R-package lme4 [40]. The response variable and the predictors maternal relatedness, rank difference, and age difference were square root transformed to normalize the distributions and then z-transformed to standardize factors.

We checked whether the assumptions of normally distributed and homogeneous residuals were fulfilled by visually inspecting a histogram and a qqplot as well as the residuals plotted against fitted values (both indicated no obvious deviations from these assumptions). Variance Inflation Factors VIF [41] were derived using the function vif of the R-package car [42] applied to a standard linear model excluding the random effects and we found all values to be below 2 suggesting that co-variation of predictors did not affect results.

The significance of the full models as compared to the null models (comprising only the intercept and the random effects) was established using a likelihood ratio test (R function anova with argument test set to "Chisq"). To achieve a more reliable p-value we fitted the models using Maximum Likelihood (rather than Restricted Maximum Likelihood; [43]. P-values for the individual effects were based on Markov Chain Monte Carlo sampling [38] and derived using the functions pvals.fnc and aovlmer.fnc of the R package languageR [44].

Because dyadic values are not independent when involving the same individual repeatedly we used a Mantel-like test of whether values in a quantitative matrix with CSI values differ between levels of a factor in a categorical matrix. The test is based on the simultaneous permutation of the rows and columns of one of the two matrices [45]. We investigated whether the strength of the social bond varies between mother-daughter, full-sister, maternal half-sister, paternal half-sister dyads and dyads of more distantly or unrelated females (Tab. 1). The procedure was written by Roger Mundry (Max Planck Institute for Evolutionary Anthropology Leipzig, Germany) and performed in R; it used the sum of the squared differences between the means per category of relatedness and the mean of these means as an overall test statistic and absolute differences between mean social bonds in two classes of relatedness for pair-wise comparisons. We ran 10.000 permutations into which we included the original data as one permutation and determined P-values as the proportion of permutations revealing a test statistic at least as large as that of the original data.

**Table 1:** Kin category, number of dyads in the group and their relatedness coefficients used in this study.

Kin category	# ofdyads	maternal	Relatednesspaternal	total
Mother-daughter	10	0.5	0	0.5
Full sisters	8	0.25	0.25	0.5
Maternal half-sisters	3	0.25	0	0.25
Paternal half-sisters*	137	<0.0625	0.25	0.25
Distant and non-kin	219	0–0.125	0	≤0.125

## Results

**Figure 1:**
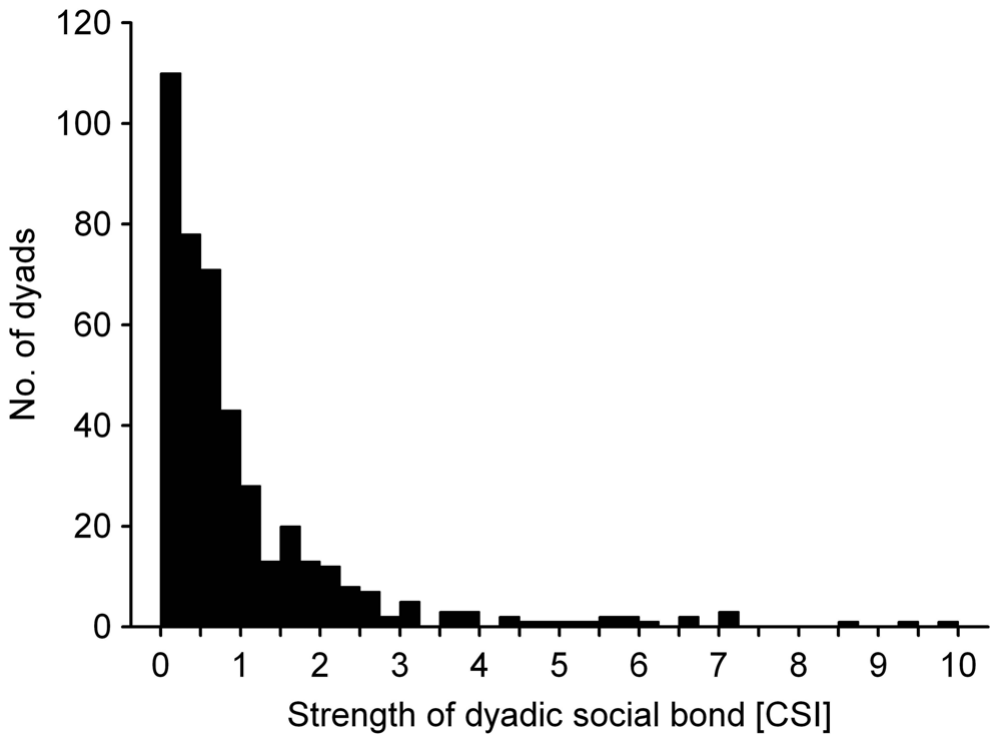
Distribution of the strength of dyadic social bonds measured as the composite sociality index (CSI) between females in a group of captive rhesus macaques.

Despite the overall high degree of relatedness in the study group and the slightly confined spatial situation, females expressed a high degree of differentiation in their social bonds. The majority of CSI scores for the 435 dyads were low or very low, with a mode of 0.61 and an interquartile range 0.24–1.19, only slightly exceeding the average of 1.0 (Fig. 1). The maximum CSI was close to 10 and 10% of dyads showed a CSI greater than 2.3.

Non-zero relatedness varied from 0.5 between mothers and daughters or full sisters and 0.004 between third degree unilateral cousins. The only paternal relation found among adult females was the paternal half-sister relation. A GLMM with maternal relatedness, rank-distance, and paternal sister-ship as predictors of the CSI was significantly different from the null model, which included only the intercept and the random effects (Chi^2^ = 46.00, df = 3, p<0.0001). Across all dyads the strength of social bonds was positively affected by the degree of maternal relatedness and by rank-similarity (Tab. 2). Paternal sister-ship explained a significant portion of the residual variance, i.e. after controlling for the effect of maternal relatedness and rank-distance paternal half-sisters had significantly closer social bonds than paternally unrelated females. We built a second model that also considered the effect of age-difference on the CSI but excluded the 10 mother-daughter dyads because of their inherently large age-difference (full model significantly different form the null model Chi^2^ = 44.15, p<0.0001). Age proximity did not have an independent effect on the strength of the social bond between two females (pMCMC = 0.8188).

**Table 2:** Predictors of the strength of social bonds (CSI) between female rhesus macaques.

	Estimate	MCMCmean	HPD95lower	HPD95upper	pMCMC
(Intercept)	0.033	0.028	−0.141	0.198	0.746
mat_rel	0.364	0.336	0.188	0.484	0.0001
pat_rel	0.323	0.314	0.183	0.456	0.0001
rank_diff	−0.183	−0.179	−0.267	−0.088	0.0002

P values for individual effects derived from Markov Chain Monte Carlo sampling (Baayen 2008). Maternal relatedness (mat_rel), paternal sistership (pat_rel), and dominance rank difference (rank_diff) had independent effects on a dyad’s CSI score.

**Figure 2:**
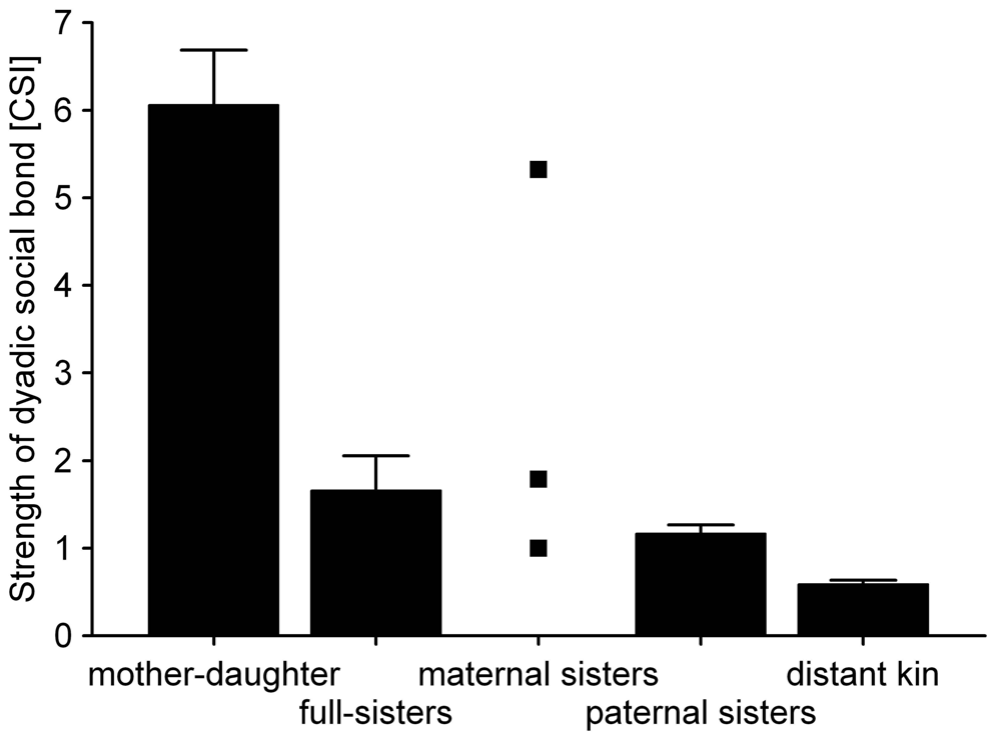
Differences in the strength of dyadic social bonds between kin categories. Bars are means and whiskers standard errors. Values for three maternal sister dyads are given as individual points. Calculated relatedness is 0.5 for mother-daughter and full-sister dyads, 0.25 for paternal, and maternal (half-)sister and below 0.125 for distant kin. For statistics see table 3.

In addition to the GLMM results comparisons between dyads with different kin relations also supported the idea that female sociality was partially guided by paternal relatedness (Fig. 2, Tab.3). Bonds between paternal sisters (mean CSI = 1.17±1.10) were stronger than those between distantly related females (coefficient of relatedness ≤0.125, mean CSI = 0.59±0.72). Paternal sisters that were peers from the same cohort (zero age difference) did not exhibit stronger social bonds than non-peer paternal sisters (mean CSI paternal sisters peers = 1.05±0.88, paternal sisters non-peers = 1.20±1.14, p-values from permutation tests = 0.46). Comparisons between paternal sister peers and unrelated peers are not possible because all peers were paternal sisters. Variation in age difference (0–5 years) was not related to variation in average CSI of paternal sisters (Spearman’s rho = 0.141, p = 0.98).

Mother-daughter dyads exhibited the closest social bonds with significantly higher CSI values (mean = 6.06±1.98) than any other kin relation analyzed (Tab. 3). Values for the three maternal sister dyads (1.05, 1.87, 5.56) were all below the mean value for mother-daughter dyads. Social bonds between full sisters were not significantly different from those between paternal half-sisters perhaps owing to the low number of full sisters in the sample (mean = 1.66±1.15; Fig 2.). Mother’s presence seemed to be an important factor influencing the bonds between sisters: bonds tended to be stronger between purely maternal as well as full sisters when they lived with their mother at the time of the study (5 dyads of 7 females, including one case of three sisters, mean CSI = 3.60) than when they had lost their mother (5 dyads of 7 other different females including one case of three sisters, mean CSI = 1.39, t = 2.26, p = 0.065). Despite the same total relatedness the bond between mother and daughter was more than three times stronger on average than between full-sisters (Fig. 2).

**Table 3:** Differences in the strength of social bonds (CSI) between kin categories.

	Full sisters	Maternal sisters	Paternal sisters	Distant and non-kin
Mother-daughter	p<0.0001	P = 0.0028	p<0.0001	p<0.0001
Full sisters		p = 0.2037	p = 0.2778	p = 0.0405
Maternal sisters			p = 0.0614	p = 0.0292
Paternal sisters				p = 0.0007

Results are reported for a Mantel-like permutation test with 10,000 permutations.

## Discussion

Our study replicated important aspects of previous studies on the factors determining the variation in the strength of social bonds. Most importantly, and despite the overall high degree of relatedness in the study group, we found that paternal sisters exhibited stronger social bonds than non-sisters in multivariate analyses controlling for the degree of maternal relatedness as well as age and rank similarity. In direct comparisons of kin categories social bonds between (maternally unrelated) paternal half-sisters were significantly stronger than those between more distantly related kin. Although the size of the effect is relatively small, these results add to the growing body of evidence for paternal kin preferences in social behavior [31] that may have far reaching consequences for the social structure of the group as a whole [46].

The fact that paternal kin is preferred is puzzling because it is still unclear how animals distinguish kin from non-kin [31]. For species with high paternity concentration in one or a few individuals, i.e. high reproductive skew, and short tenures age similarity has been proposed as a cue to paternal relatedness [36]. While animals in some populations may use age similarity as an approximation [33] it may not be a reliable indicator of paternal relatedness in others; despite considerable male reproductive skew chimpanzee infants at Ngogo, Uganda can be as genetically related to an individual within their own cohort as to an infant from a previous/future cohort [34]. The strict breeding regimen in our study group creates a similar situation to that in Ngogo chimpanzees in the sense that the number of paternal sisters across cohorts decreases only slowly with increasing age difference; there were 42 paternal sisters from the same cohort and 30 paternal sisters in the group that were three years apart in age. All females from the same cohort were paternal sisters, i.e. paternally related at r = 0.25. Females born 1 year apart were still related paternally at r = 0.19 on average, i.e. they had a 75% chance of being paternal sisters. The probability of non-peers aged 1–6 years apart was still as high as 0.49 which may explain why age similarity did not affect sociality in this study. Unlike in a previous study on rhesus macaques [24] familiarity did not add to paternal kin discrimination.

Phenotypic matching may play a crucial role in paternal kin discrimination; e.g. rhesus macaque facial geometry carries information about paternal relatedness [47], [48] that can be picked up by conspecifics [48]. In vertebrates kin may also be discriminated by odor [49], [50] or auditory cues [51]. Whether the lack of preferential affiliation with paternal kin in some populations [20], [22], [34], [52] results from the inability to differentiate paternal kin from non-kin remains unclear. On the one hand paternal kin discrimination may not be selected for if large benefits can be reaped from indiscriminate low cost investments [53] which requires that females do not compete in a zero sum game and that the benefits of cooperating are not counterbalanced by kin competition. Perfect paternal kin discrimination may even be selected against by male infanticide [54]. On the other hand paternal kin discrimination may simply not be used to channel affiliative behavior as long as enough maternal relatives are available as partners. Capuchin monkey females on average have 3.3 close maternal kin available and do not prefer paternal kin [52]. Baboons at Amboseli turn to paternal sisters only when they are devoid of close maternal kin [27]. Our result that social bonds among paternal half-sisters are weaker than mother daughter bonds and full sister bonds (the latter difference is in the right direction but not significant perhaps due to small sample size) support this claim. More data on a more diverse range of societies is needed to tackle these questions from a comparative perspective.

Maternal relatedness was a good predictor of the strength of social bonds between rhesus macaque females. The social bond between mothers and their offspring if carried into adulthood was by far the strongest [5]. The fact that mother-daughter bonds were almost three times as strong as those between full sisters may suggest that females discount paternal relatedness, i.e. they value paternal relatedness less than maternal relatedness when they allocate their affiliative efforts. Both kin categories are related at r = 0.5 but 100% of the genes shared by common descent are of maternal origin in the mother-daughter dyad while 50% are of paternal origin in the case of full sisters. Few other studies included enough full sister dyads for meaningful comparison to mother daughter dyads but a similar argument can be made for the following comparisons.

In both maternal half-sister dyads and paternal half-sister dyads the same proportion of genetic material is shared by common descent but paternal genes seem to be discounted because social bonds among maternal sisters are frequently much stronger than those between paternal sisters [24], [27], [52]. It seems likely that these discounting effects result from variation in accuracy of relationship assessment. In mammals the relationship between mother and daughter is most easily assessed by the individual itself. Any other relationship, e.g. the one between maternal sisters, needs to be assessed more indirectly which may cause inaccuracy. This inaccuracy may be even larger for paternal than for maternal kin. Several modes of information (familiarity, behavioral, olfactory, auditory and visual cues) can be integrated into an assessment of maternal relatedness. Familiarity through bonding with the same third individual is less pronounced among more lateral and especially among paternal kin [25], [31]. As a consequence, the strength of kin selection may vary between descendent and lateral kin as well as between maternal and paternal kin.

Our results also add to the discussion on the role of mothers in maintaining the relationships between sisters. Female baboons at Amboseli form stronger bonds with their sisters if their mother is no longer present in the group [27]. In contrast, rhesus macaque maternal or full sisters in our study were more closely bonded if their mother was still alive. While the first finding suggests that upon the death of their preferred partner, i.e. the mother, females turn to their sisters as the next best partner our finding suggests that sisters are kept close by the existence of their mother. The clustering of sisters around their mother is not a mere spatial effect because sisters with a mother in the group do not only spend more time in close proximity (which could be mediated by each of them being close to a third female, i.e. the mother) but they also groom each other more often than sisters that lost their mother (6.8 times versus 3.9 times across the study period on average). As a consequence and conditional upon the availability of appropriate numbers of descendant kin functional matrilines may split in two upon the death of the matriarch.

The threshold for nepotistic biases in affiliative behavior was realized in the unilateral aunt niece relationship with only marginally closer bonds than those among non-kin (difference between means 0.165, p = 0.46). This is in general agreement with previous studies showing that kin preferences fade below r-values of 0.25 for lateral and 0.125 for descendant kin [25], [27], [29]. Collectively these results suggest that, especially in the absence of close descendent kin, close paternal kin may play an important role in structuring social networks.
